# Supplementary material for: Global and local mobility as a barometer for COVID-19 dynamics
Source: medRxiv. 2020 Aug 11:2020.06.13.20130658. Preprint. [Version 2] doi: 10.1101/2020.06.13.20130658 (PMC7430597; doi:10.1101/2020.06.13.20130658)
Supplement: 1 [file NIHPP2020.06.13.20130658-supplement-1.pdf]

medRxiv preprint doi: https://doi.org/10.1101/2020.06.13.20130658.this version posted August 11, 2020. The copyright holder for this preprint (which was not certified by peer review) is the author/funder, who has granted medRxiv a license to display the preprint in perpetuity.
It is made available under a CC-BY 4.0 International license .

Supplementary Material

Local epidemiology modeling - SEIIRD model

To illustrate that our method is robust to disease parameters beyond the classical SEIR model, we expand the SEIR model for the local epidemiology of COVID-19 from Eq. (1) to an SEIIRD model with six compartments, the susceptible, exposed, detected infectious, hidden infectious, recovered, and deceased populations. These six compartments are governed by the following set of ordinary differential equations,

$$\begin{aligned}
\dot{S} &= -\beta(t)\,S\,[I_{\rm s}+I_{\rm a}] \\
\dot{E} &= +\beta(t)\,S\,[I_{\rm s}+I_{\rm a}] - \alpha\,E \\
\dot{I}_{\rm d} &= +\nu_{\rm d}\,\alpha\,E - [1-\nu_{\rm f}]\,\gamma\,I_{\rm d} - \nu_{\rm f}\,\sigma\,I_{\rm d} \\
\dot{I}_{\rm h} &= +[1-\nu_{\rm d}]\,\alpha\,E - \gamma\,I_{\rm h} \\
\dot{R} &= +\gamma\,I_{\rm h} + [1-\nu_{\rm f}]\,\gamma\,I_{\rm d} \\
\dot{D} &= +\nu_{\rm f}\,\sigma\,I_{\rm d}
\end{aligned} \tag{S.1}$$

The transition rates between the compartments, $\beta(t)$, α, γ, and σ are inverses of the contact period $B(t) = 1/\beta(t)$, the latent period $A = 1/\alpha$, the infectious period $C = 1/\gamma$, and the survival period $S_{\rm D} = 1/\sigma$. The SEIIRD model splits the initial infectious group of the SEIR model in Eq. (1) into a detected group, $I_{\rm d} = \nu_{\rm d}\,I$, and a hidden undetected group, $I_{\rm h} = \nu_{\rm h}\,I$, where $\nu_{\rm d}$ is the detection fraction and $\nu_{\rm h} = 1 - \nu_{\rm d}$. From the detected group $I_{\rm d}$, individuals can transition into the recovered group R at a fraction $[1-\nu_{\rm f}]$ or into the deceased group D at a fraction $\nu_{\rm f}$, where $\nu_{\rm f}$ is the case fatality rate. We interpret the latent, infectious and survival periods A, C, and $S_{\rm D}$ as disease-specific and static, and the contact period $B(t)$ as behavior specific and dynamic. Using the dynamic contact period $B(t)$, we calculate the effective reproduction number $R(t) = C/B(t)$ to quantify the outbreak dynamics.

We need to estimate a set of 15 parameters including a set of eight parameters for the SEIIRD model, $\boldsymbol{\theta}_{\rm SEIIRD} = \{\,\alpha,\,\gamma,\,\sigma,\,\nu_{\rm d},\,\nu_{\rm f},\,E_0,\,I_{\rm d,0},\,I_{\rm h,0}\}$, and a set of seven parameters for the semi-parametric model, $\boldsymbol{\theta}_{\rm Rt} = \{\,t^*, T,\,\eta^2,\,\ell^2, k, m, \boldsymbol{\delta}\}$. As before, we estimate the model parameter set $\boldsymbol{\theta}^* = \boldsymbol{\theta}_{\rm SEIIRD} \cup \boldsymbol{\theta}_{\rm Rt}$ using Bayesian inference with Markov Chain Monte Carlo sampling. We fix the latency and infectious periods to $A = 2.5$ days and $C = 6.5$ days. In contrast to the SEIR model, we now fit two data sets simultaneously, the new daily detected cases, $\Delta\hat{I}_{\rm d}(t) = \hat{I}_{\rm d}(t_{\rm n+1}) - \hat{I}_{\rm d}(t_{\rm n})$, and the daily new deaths, $\Delta\hat{D}(t) = \hat{D}(t_{\rm n+1}) - \hat{D}(t_{\rm n})$, which we extract as the differences between the today's and yesterday's confirmed cases and deaths (*2*) and smoothen using a seven-day moving average on the data. For both fits, we adopted a Student's t-distribution for the likelihood between the new daily reported data, $\Delta\hat{I}_{\rm d}(t)$ and $\Delta\hat{D}(t)$, and the model predictions, $\Delta I_{\rm d}(t,\boldsymbol{\theta}^*)$ and $\Delta D(t,\boldsymbol{\theta}^*)$, with new-case- and new-deaths-number-dependent widths (*1*),

medRxiv preprint doi: https://doi.org/10.1101/2020.06.13.20130658.this version posted August 11, 2020. The copyright holder for this preprint (which was not certified by peer review) is the author/funder, who has granted medRxiv a license to display the preprint in perpetuity.
It is made available under a CC-BY 4.0 International license .

$$
\begin{aligned}
p\left(\Delta\hat{I}_{\rm d}(t)|\boldsymbol{\theta}^*\right) &\sim \text{StudentT}_{\nu=4}\left(\text{mean}=\Delta I_{\rm d}(t,\boldsymbol{\theta}^*),\text{width}=\sigma_{\rm I}\sqrt{\Delta \text{I}_{\rm d}(\text{t},\boldsymbol{\theta}^*)}\right),\\
p\left(\Delta\hat{D}(t)|\boldsymbol{\theta}^*\right) &\sim \text{StudentT}_{\nu=4}\left(\text{mean}=\Delta D(t,\boldsymbol{\theta}^*),\text{width}=\sigma_{\rm D}\sqrt{\Delta \text{D}(\text{t},\boldsymbol{\theta}^*)}\right),
\end{aligned}
\qquad \text{(S.2)}
$$

where σ_I and σ_D represent the widths of the likelihoods $p(\Delta\hat{I}_{\rm d}(t)|\boldsymbol{\theta}^*)$ and $p(\Delta\hat{D}(t)|\boldsymbol{\theta}^*)$ between the daily new reported cases and deaths $\Delta\hat{I}_{\rm d}$ and $\Delta\hat{D}$ and the associated modeled new cases and deaths $\Delta I_{\rm d}$ and ΔD. Again, we apply Bayes' rule to obtain the posterior distributions of the parameters on the basis of the prior distributions specified in Table S3, and the reported cases and deaths themselves, which we infer approximately by employing the NO-U-Turn sampler (NUTS) (*4*) implementation of the Python package PyMC3 (*6*).

Fig. S1 shows the reported and simulated cases and deaths across Europe. The hierarchical SEIIRD model learns the time-varying effective reproduction number $R(t)$ from both the reported cases and deaths in Fig. S1 (a) for varying adaptation times t^*. The learnt survival period is $S_{\rm D} = 6.62 \pm 0.14$ days. The adaptation time distribution in Fig. S1 (b) indicates an adaptation time of $t^* = 16.6 \pm 2.8$ days. The box plots in Figs. S1 (c & d) show the country-specific case fatality rates $\nu_{\rm f}$ and detection fractions $\nu_{\rm d}$. These results suggest that our method is not only applicable to the classical SEIR model but extends equally to more sophisticated models like the SEIIRD model with additional hidden and deceased compartments, and can not only fit the reported case data, but also simultaneously reported cases and deaths.

Sensitivity with respect to date of reporting

In our main study, we interpret the daily reported case number $\Delta\hat{I}(t)$ from a central European data base (*2*) as the infectious population and fit this case number against the daily change of the infectious population $\Delta I(t,\boldsymbol{\theta})$ of our SEIR model. Arguably, the "date of reporting" can mean different things for different countries, and it can vary hugely between infection, symptom onset, testing, positive confirmed, and reporting. This implies that our time delay between mobility and reproduction is highly sensitive to the local testing logistics.

Table S1 illustrates difference between symptom onset and reporting for four representative countries (*3*). For example, in Germany, the "date of reporting" represents the date at which a sample swab is sent to laboratory testing, although the actual date at which authorities are notified will be several days later. To illustrate the difference between symptom onset and reporting, we perform the same SEIR model analysis based on two different reported data sets $\hat{I}(t)$, symptom onset data from the Robert Koch Institute (*5*) and reporting data from European Centre for Disease Prevention and Control (*2*), which includes the data that we used throughout this study.

medRxiv preprint doi: https://doi.org/10.1101/2020.06.13.20130658.this version posted August 11, 2020. The copyright holder for this preprint
(which was not certified by peer review) is the author/funder, who has granted medRxiv a license to display the preprint in perpetuity.
It is made available under a CC-BY 4.0 International license .

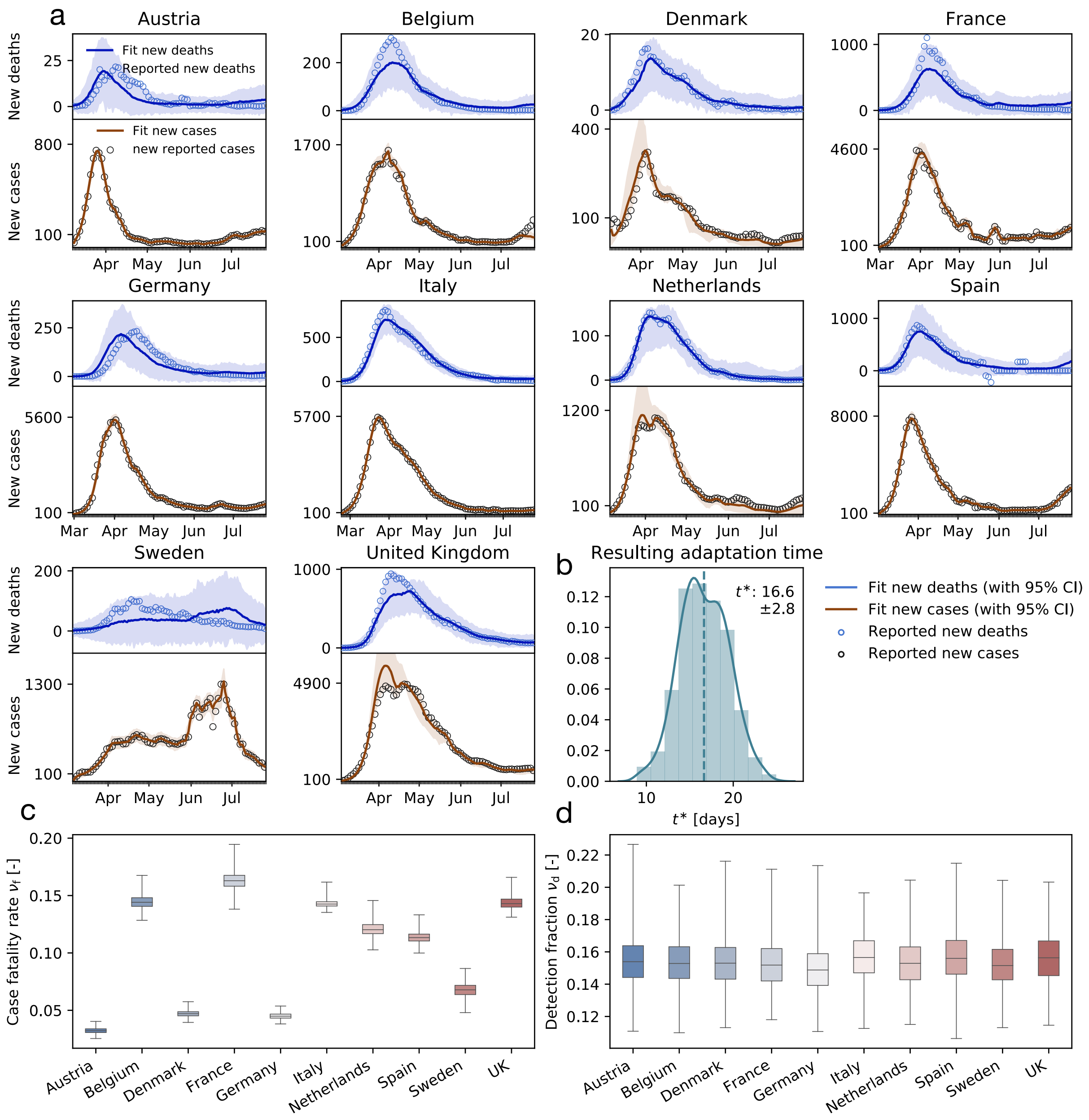

Figure S1: **Reported and simulated cases and deaths across Europe.** (a) The hierarchical SEIIRD model learns the time-varying effective reproduction number $R(t)$ from the reported cases (black circles) and deaths (blue circles) for varying adaptation times t^*; (b) adaptation time distribution indicates an adaptation time of $t^* = 16.6 \pm 2.8$ days; (c) box plots of country-specific case fatality rates ν_f; (d) box plots of country-specific detection fractions ν_d.

Figure S2 illustrates the learned time-varying effective reproduction numbers for both symptom onset and reporting data, and the model fit of the simulated cases to the two data sets. The time shift in the two data sets results in a time shift of the model

medRxiv preprint doi: https://doi.org/10.1101/2020.06.13.20130658.this version posted August 11, 2020. The copyright holder for this preprint (which was not certified by peer review) is the author/funder, who has granted medRxiv a license to display the preprint in perpetuity.
It is made available under a CC-BY 4.0 International license .

Table S1: **Time delay between symptoms onset and reporting (*3*).**

**Countries**	**Median** [days]	**Mean** [days]
EU/EEA and UK	5	7
Estonia	5	6
Luxembourg	5	6
Romania	5	7
United Kingdom	4	4

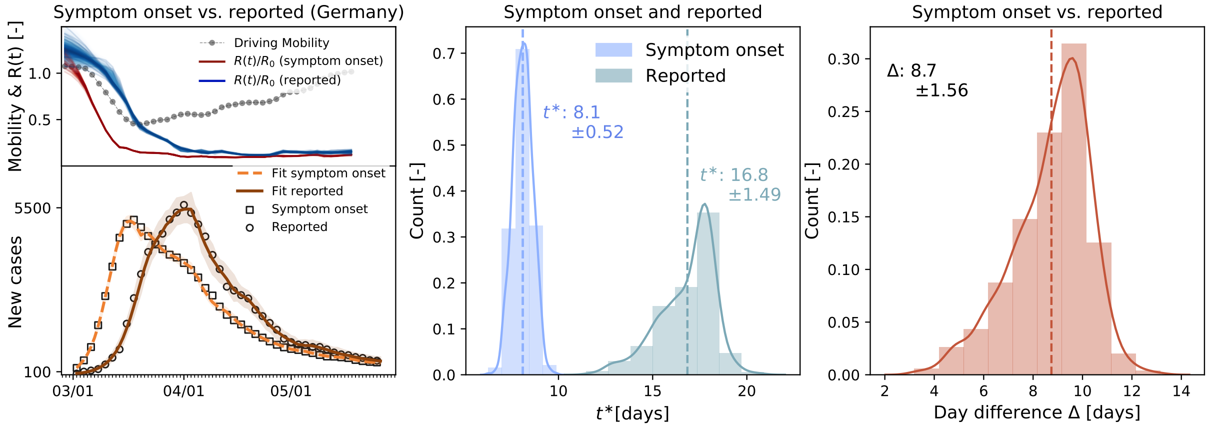

Figure S2: **Local mobility, reproduction number, and reported cases in Germany.** The model learns the time-varying effective reproduction number for symptom onset (red curve) from the symptom onset cases (black boxes) and simulated cases (orange curve) and for reporting (blue curve) from the reported cases (black circles) and simulated cases (brown curve). Distributions of adaptation time t^* for symptom onset (blue) and reported data (green), and of difference between symptom onset and reported data (red).

fit, and with it, a time shift in the effective reproduction number curves. The resulting adaptation time distributions vary between $t^* = 8.10 \pm 0.52$ days for the system onset data and $t^* = 16.80 \pm 1.49$ days for the reported data with a mean difference of 8.7 days. This study highlights the sensitivity of the data to the reporting logistics. Unfortunately, system onset data were not available for all countries in the European Union, and we can only show the sensitivity here, rather than using system onset data throughout our entire study, which would have been more unified and accurate.

Forecasting

Fig. S3 compares the results of a two-week forecasting with different training sets for all ten European countries. Fig. S3 (a) uses the full available data set, whereas Fig. S3 (b) uses only a reduced data set beginning on April 1, 2020 to train the model.

medRxiv preprint doi: https://doi.org/10.1101/2020.06.13.20130658.this version posted August 11, 2020. The copyright holder for this preprint
(which was not certified by peer review) is the author/funder, who has granted medRxiv a license to display the preprint in perpetuity.
It is made available under a CC-BY 4.0 International license .

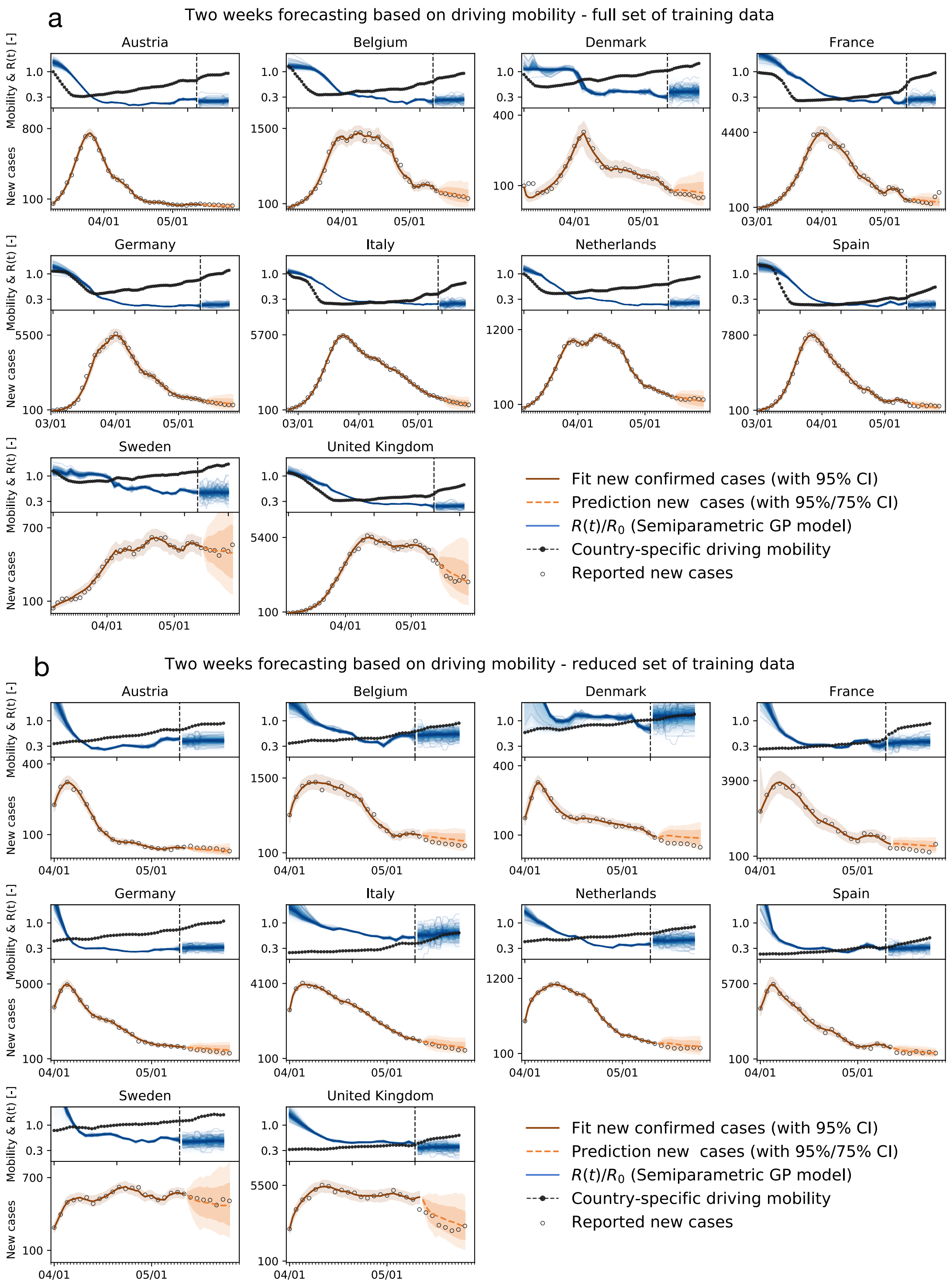

Figure S3: **Local mobility, reproduction number, reported cases, and forecasting with different training sets.** (a) Two-week forecast with training on the full available data set; and (b) Two-week forecast with training on reduced data set beginning on April 1, 2020.

medRxiv preprint doi: https://doi.org/10.1101/2020.06.13.20130658.this version posted August 11, 2020. The copyright holder for this preprint (which was not certified by peer review) is the author/funder, who has granted medRxiv a license to display the preprint in perpetuity.
It is made available under a CC-BY 4.0 International license .

Network modeling

Fig. S4 shows results of the network model including both global and local mobility. The global mobility represents the air traffic data in each country, the local mobility represents the driving mobility.

Priors on the model parameters

Tables S2 and S3 summarize the priors on our the semi-parametric network model parameters and on our SEIIRD model parameters.

Table S2: **Priors on the semi-parametric (network) model parameters.**

Parameter	Variables	Prior distributions
R_0	basic reproduction	Normal(2, 1.5)
σ_{R_0}	std basic reproduction	HalfNormal(1.5)
t^*	adaptation time	Normal(14, 14)
σ_{t^*}	std adaptation time	HalfNormal(15)
T	transition time	LogNormal($\log(3)$, 0.8)
σ_T	std transition time	HalfNormal(0.8)
ℓ^2	hyperparameter	Gamma(2, 0.1)
η	hyperparameter	HalfCauchy(0.5)
k	growth rate	Normal(1, 1)
m	offset	Normal(1, 1)
$\boldsymbol{\delta}$	rate adjustment	Normal(0, 2, shape=S_{p})
I_0	initial infected	LogNormal($\log[\Delta\hat{I}(t=0)]$, 1.0)
E_0	initial exposed	LogNormal($\log[\Delta\hat{I}(t=3)]$, 1.0)
ϑ_0	travel coefficient	Normal(0.4,0.3)
σ_{I}	likelihood width	HalfCauchy($\beta = 1$)

References

1. J. Dehning, J. Zierenberg, F.P Spitzner, M. Wibral, J.P. Neto, M. Wilczek, V. Priesemann. Inferring COVID-19 spreading rates and potential change points for case number forecasts. Science (2020) doi: 10.1126/science.abb9789

2. European Centre for Disease Prevention and Control - Covid-19 situation update worldwide. `https://www.ecdc.europa.eu/en/geographical-distribution-2019-ncov-cases`. accessed: 2020-08-04

medRxiv preprint doi: https://doi.org/10.1101/2020.06.13.20130658.this version posted August 11, 2020. The copyright holder for this preprint
(which was not certified by peer review) is the author/funder, who has granted medRxiv a license to display the preprint in perpetuity.
It is made available under a CC-BY 4.0 International license .

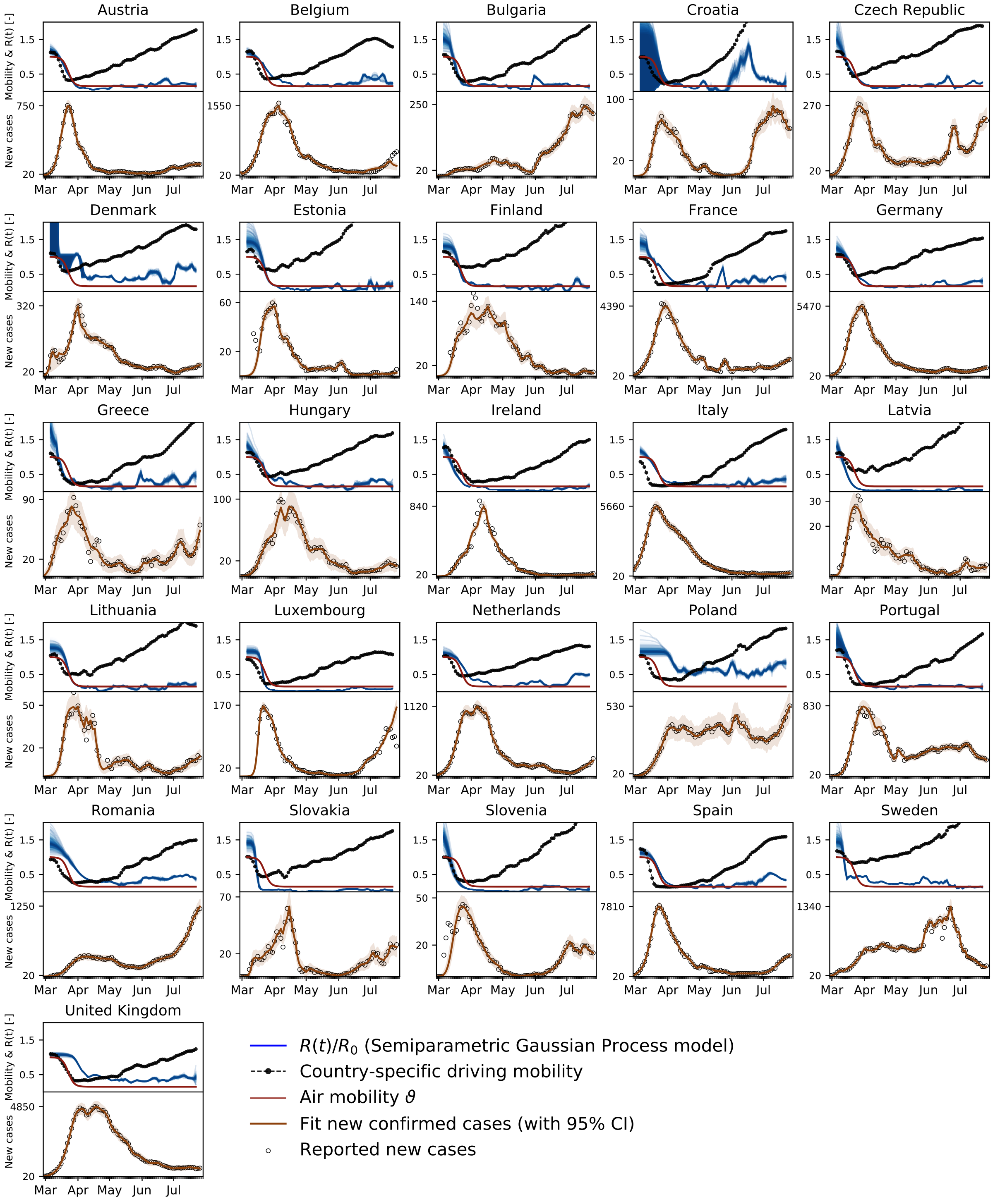

Figure S4: **Global and local mobility, reproduction number, and reported cases across Europe using the network model.** The model learns the time-varying effective reproduction number R(t) (blue curves) from the reported cases (black circles) and simulated new cases (orange curves). Global mobility (red curves) and local mobility (black curves) highlight the reduction in air traffic and driving mobility.

medRxiv preprint doi: https://doi.org/10.1101/2020.06.13.20130658.this version posted August 11, 2020. The copyright holder for this preprint (which was not certified by peer review) is the author/funder, who has granted medRxiv a license to display the preprint in perpetuity.
It is made available under a CC-BY 4.0 International license .

Table S3: **Priors on the SEIIRD model parameters.**

Parameter	Variables	Prior distributions
R_0	basic reproduction	Normal(2, 1.5)
σ_{R_0}	std basic reproduction	HalfNormal(1.5)
t^*	adaptation time	Normal(14, 14)
σ_{t^*}	std adaptation time	HalfNormal(15)
T	transition time	LogNormal($\log(3)$, 0.8)
σ_T	std transition time	HalfNormal(0.8)
ℓ^2	hyperparameter	Gamma(2, 0.1)
η	hyperparameter	HalfCauchy(0.5)
k	growth rate	Normal(1, 1)
m	offset	Normal(1, 1)
$\boldsymbol{\delta}$	rate adjustment	Normal(0, 2, shape=S_{p})
S_{D}	survival period	Normal(6.5,1)
ν_{d}	detection fraction	LogNormal($\log[0.15]$, 0.1)
ν_{f}	case fatality rate	LogNormal($\log[0.01]$, 1.0)
$I_{\mathrm{d},0}$	initial detected infected	LogNormal($\log[\Delta\hat{I}(t=0)]$, 1.0)
$I_{\mathrm{h},0}$	initial hidden infected	Deterministic($[1-0.15]/0.15\, I_{\mathrm{d},0}$)
E_0	initial exposed	LogNormal($\log[\Delta\hat{I}(t=3)]$, 1.0)
σ_{I}	likelihood width	HalfCauchy($\beta=1$)
σ_{D}	likelihood width	HalfCauchy($\beta=1$)

3. European Centre for Disease Prevention and Control - Covid-19 surveillance report. https://covid19-surveillance-report.ecdc.europa.eu/#5_risk_groups_most_affected. accessed: 2020-08-04

4. M.D. Hoffman, A. Gelman. The No-U-Turn sampler: adaptively setting path lengths in Hamiltonian Monte Carlo. Journal of Machine Learning Research (2014), 15(1), 1593-1623

5. Robert Koch Institute. COVID-19-Dashboard. https://experience.arcgis.com/experience/. accessed: 2020-08-04

6. J. Salvatier, T.V. Wiecki, C. Fonnesbeck. Probabilistic programming in Python using PyMC3. Peer Journal Computational Science 2 (2016) e55
